# Supplementary material for: NICU Admissions for Meconium Aspiration Syndrome before and after a National Resuscitation Program Suctioning Guideline Change
Source: Children (Basel). 2019 May 7;6(5):68. doi: 10.3390/children6050068 (PMC6560382; doi:10.3390/children6050068)
Supplement: Supplementary file 1 [file children-06-00068-s001.pdf]

Table S1. Participating Centers

Huntsville Hospital	Alabama
Baptist Medical Center East	Alabama
St. Vincent Birmingham	Alabama
The Children's Hospital at Providence, Alaska	Alaska
St. Joseph's Hospital and Medical Center	Arizona
Cardon Children's Medical Center	Arizona
Banner Thunderbird Medical Center	Arizona
Banner Estrella Medical Center	Arizona
Abrazo Arrowhead Campus	Arizona
Arizona Children's Center Maricopa Integrated Health	Arizona
Flagstaff Medical Center	Arizona
Willow Creek Women's Hospital	Arkansas
University of Arkansas for Medical Sciences	Arkansas
Mercy Hospital Fort Smith	Arkansas
Providence Tarzana Medical Center	California
Providence Little Company of Mary Medical Center	California
Rocky Mountain Hospital for Children at Presbyterian/Saint Luke’s	Colorado
St. Mary's Hospital and Medical Center	Colorado
Swedish Medical Center	Colorado
Poudre Valley Hospital	Colorado
Good Samaritan Medical Center	Colorado
Denver Health Medical Center	Colorado
Stamford Hospital	Connecticut
Danbury Hospital	Connecticut
Yale-New Haven Children's Hospital	Connecticut
Connecticut Children's NICU at UCONN Health Center	Connecticut
Norwalk Hospital	Connecticut
The Hospital of Central Connecticut	Connecticut
Greenwich Hospital	Connecticut

Washington Hospital Center	District of Columbia
Joe DiMaggio Children's Hospital	Florida
Florida Hospital for Children	Florida
Sacred Heart Health System	Florida
Baptist Children's Hospital	Florida
St. Joseph's Children's Hospital	Florida
Women's Center at Florida Hospital - Tampa	Florida
UF Shands Hospital Gainesville	Florida
UF Health Jacksonville	Florida
North Florida Regional Medical Center, Inc.	Florida
Gwinnett Hospital System	Georgia
The Medical Center at Columbus Regional	Georgia
Piedmont Rockdale Hospital	Georgia
Hamilton Medical Center	Georgia
Floyd Medical Center	Georgia
Kapiolani Medical Center for Women & Children	Hawaii
St. Luke's Regional Medical Center	Idaho
St. Luke's Magic Valley	Idaho
Eastern Idaho Regional Medical Center	Idaho
Advocate Children's Hospital - Park Ridge	Illinois
Children’s Hospital of Illinois at OSF St. Francis Medical Center	Illinois
St. John's Hospital	Illinois
Carle Foundation Hospital	Illinois
Rockford Memorial Hospital	Illinois
University of Illinois at Chicago	Illinois
Advocate Children's Hospital - Oak Lawn	Illinois
Advocate Illinois Masonic Medical Center	Illinois
Edward Hospital and Health Services	Illinois
Rush Copley Medical Center	Illinois
Advocate Good Samaritan Hospital	Illinois

Adventist Hinsdale Hospital	Illinois
St. Alexius Medical Center	Illinois
Provena Saint Joseph Medical Center	Illinois
St. Joseph Hospital Chicago	Illinois
Memorial Hospital of Carbondale	Illinois
St. Vincent Women's Hospital	Indiana
Dupont Hospital	Indiana
Franciscan Health Lafayette	Indiana
St. Vincent Evansville	Indiana
Lutheran Hospital of Indiana	Indiana
Women's Hospital	Indiana
St. Joseph Hospital	Indiana
St. Vincent Carmel Hospital	Indiana
Franciscan St. Francis Health	Indiana
Blank Children's Hospital	Iowa
Genesis Medical Center	Iowa
St. Luke's Hospital	Iowa
St. Luke's Regional Medical Center	Iowa
Covenant Medical Center	Iowa
Wesley Medical Center	Kansas
Via Christi Hospitals Wichita	Kansas
University of Kansas Hospital Authority	Kansas
Overland Park Regional Medical Center	Kansas
Shawnee Mission Medical Center	Kansas
Kentucky Children's Hospital	Kentucky
Norton Children's Hospital	Kentucky
University of Louisville Hospital	Kentucky
Norton Women's and Children's Hospital	Kentucky
The Medical Center NICU Bowling Green	Kentucky
King's Daughters Medical Center	Kentucky

Methodist Hospital	Kentucky
Jennie Stuart Medical Center	Kentucky
Baptist Health Madisonville	Kentucky
Frankfort Regional Medical Center	Kentucky
Baptist Health Lexington	Kentucky
Owensboro Health Regional Hospital	Kentucky
Baptist Health Paducah	Kentucky
The Women's Hospital at St. Joseph East	Kentucky
St. Elizabeth Healthcare	Kentucky
Woman's Hospital	Louisiana
Willis Knighton South	Louisiana
Christus Lake Area Hospital	Louisiana
Christus St. Frances Cabrini Hospital	Louisiana
West Jefferson Medical Center	Louisiana
Ochsner Medical Center WestBank	Louisiana
Lake Charles Memorial Hosp for Women	Louisiana
Touro Infirmary	Louisiana
CHRISTUS Highland	Louisiana
Ochsner Medical Center Baton Rouge	Louisiana
Terrebonne General Medical Center	Louisiana
Eastern Maine Medical Center	Maine
Frederick Memorial Hospital	Maryland
Johns Hopkins Hospital	Maryland
Prince George's Hospital Center	Maryland
UMass Memorial Healthcare	Massachusetts
Steward St. Elizabeth's Medical Center	Massachusetts
Baystate Medical Center	Massachusetts
Tufts Medical Center	Massachusetts
Beth Israel Deaconess Medical Center	Massachusetts
Brigham and Women's Hospital	Massachusetts

Massachusetts General Hospital for Children	Massachusetts
South Shore Hospital	Massachusetts
Boston Medical Center	Massachusetts
DeVos Children's, Spectrum Health	Michigan
Sparrow Hospital	Michigan
U. of MI, CS Mott Children's, Brandon NICU	Michigan
Children's Hospital at Bronson	Michigan
Munson Medical Center	Michigan
UPHS - Marquette	Michigan
Children's Hospitals and Clinics - Minneapolis	Minnesota
University of MN Masonic Children's Hospital	Minnesota
Children's Hospitals and Clinics - St Paul	Minnesota
Hennepin County Medical Center	Minnesota
Merit Health River Oaks	Mississippi
North Mississippi Medical Center	Mississippi
Forrest General Hospital	Mississippi
Mississippi Baptist Health Systems	Mississippi
Merit Health Wesley	Mississippi
St. Dominic, Jackson Memorial Hospital	Mississippi
Merit Health Woman's Hospital	Mississippi
Children's Hospital at U. of Mississippi Health Care	Mississippi
SSM Health Cardinal Glennon Children's Hospital	Missouri
St. Luke's Hospital	Missouri
Women's & Children's Hosp, U. of MO	Missouri
St. Louis Children's Hospital	Missouri
Centerpoint Medical Center	Missouri
Research Medical Center	Missouri
Cox Health Neonatology	Missouri
Mercy Kids Springfield	Missouri
St. Vincent Hospital & Health Center	Montana

Benefis Healthcare	Montana
Billings Clinic	Montana
CHI Health Bergan Mercy Medical Center	Nebraska
Nebraska Medical Center	Nebraska
Bryan Medical Center	Nebraska
Good Samaritan Hospital Kearney	Nebraska
Methodist Women's Hospital	Nebraska
Children's Hospital of Nevada at UMC	Nevada
St. Rose Dominican Hospital Siena Campus	Nevada
Elliot Hospital	New Hampshire
Monmouth Medical Center	New Jersey
Hackensack University Medical Center	New Jersey
Robert Wood Johnson University Hospital	New Jersey
Community Medical Center Toms River	New Jersey
Mountainside Hospital	New Jersey
Atlanticare Regional Medical Center	New Jersey
Shore Medical Center	New Jersey
Inspira Health Network	New Jersey
UNM School of Medicine Dept. of Pediatrics	New Mexico
Lovelace Women's Hospital	New Mexico
St. Joseph's Health Center	New York
Lenox Hill Hospital	New York
Richmond University Medical Center	New York
Weiler Hospital Montefiore	New York
Columbia University Medical Center	New York
Maimonides Medical Center	New York
North Shore University Hospital	New York
Cohen Children's Medical Center of New York	New York
Mt. Sinai Kravis Children's Hospital, The	New York
Montefiore Medical Center - Wakefield Division	New York

Vidant Medical Center	North Carolina
Cone Health Women's Hospital	North Carolina
NHRMC Betty H.Cameron Women & Children's Hospital	North Carolina
Forsyth Memorial Hospital	North Carolina
Cape Fear Valley Medical Center	North Carolina
WakeMedical Center	North Carolina
Mission Children's Hospital	North Carolina
FirstHealth Moore Regional Hospital	North Carolina
Catawba Valley Medical Center	North Carolina
Onslow Memorial Hospital	North Carolina
Rex Hospital	North Carolina
Jeff Gordon Children's Hospital at CMC	North Carolina
Sanford Medical Center Fargo	North Dakota
Sanford Bismarck Medical Center	North Dakota
Essentia Health	North Dakota
Trinity Hospital	North Dakota
Miami Valley Hospital	Ohio
Aultman Hospital	Ohio
NCH at Riverside Methodist Hospital	Ohio
NCH at Grant Medical Center	Ohio
Akron Children's NICU at St. Elizabeth - Boardman	Ohio
NCH at Doctor's Hospital West	Ohio
Cleveland Clinic Foundation, The	Ohio
University Hospital, Cincinnati	Ohio
NCH NICU at Ohio State Medical Center	Ohio
NCH at Dublin Methodist Hospital	Ohio
Kettering Medical Center	Ohio
NCH NICU at Mount Carmel St. Ann's Hospital	Ohio
Henry Zarrow Neonatal Intensive Care Unit	Oklahoma
O.U. Health Sciences Center	Oklahoma

St. John Medical Center	Oklahoma
Randall Children's Hospital at Legacy Emanuel	Oregon
Providence St. Vincent Medical Center	Oregon
Rogue Regional Medical Center	Oregon
Sacred Heart Medical Center	Oregon
St. Charles Health Care	Oregon
Oregon Health and Science University	Oregon
Salem Hospital	Oregon
St. Luke's University Hospital	Pennsylvania
Thomas Jefferson University Hospital	Pennsylvania
Hahnemann University Hospital	Pennsylvania
St. Luke's Allentown Campus	Pennsylvania
St. Vincent Health Center	Pennsylvania
Penn Highlands DuBois	Pennsylvania
Hamot Medical Center	Pennsylvania
Moses Taylor Hospital	Pennsylvania
Ephrata Community Hospital	Pennsylvania
Riddle Hospital	Pennsylvania
St. Mary Medical Center	Pennsylvania
Einstein Medical Center Montgomery	Pennsylvania
Children's Hospital of Greenville	South Carolina
Medical University of South Carolina	South Carolina
McLeod Regional Medical Center	South Carolina
Palmetto Health Richland	South Carolina
Palmetto Health Baptist Women's & Neonatal Services	South Carolina
Summerville Medical Center	South Carolina
Trident Medical Center	South Carolina
Avera McKennan	South Dakota
Boekelheide NICU at Sanford Health	South Dakota
Rapid City Regional Hospital	South Dakota

University of Tennessee Medical Center	Tennessee
Baptist Memorial Hospital for Women	Tennessee
Niswonger Children's Hospital	Tennessee
Monroe Carell Jr. Children's Hospital Vanderbilt	Tennessee
Maury Regional Medical Center	Tennessee
Harris Methodist Fort Worth Hospital	Texas
Texas Tech University Health Science Center	Texas
McLane Children's Hospital	Texas
John Peter Smith Hospital	Texas
Memorial Hermann Southwest	Texas
Medical City Plano	Texas
Corpus Christi Medical Center	Texas
Christus Good Shepherd Medical Center	Texas
Children's Memorial Hermann Hospital	Texas
Texas Health Presbyterian Hospital Plano	Texas
Texas Children's Hospital, Baylor College of Medicine	Texas
Texas Health Arlington Memorial Hospital	Texas
Memorial Hermann Memorial City Medical Center	Texas
Baylor All Saints Medical Center	Texas
Woman's Hospital of Texas, The	Texas
Memorial Hermann The Woodlands	Texas
Valley Regional Medical Center Texas	Texas
Medical City Lewisville	Texas
Memorial Hermann Southeast	Texas
Christus Trinity Mother Frances Health System	Texas
Covenant Women and Children's Hospital	Texas
St. Luke's The Woodlands Hospital	Texas
Methodist Willowbrook Hospital	Texas
Lyndon B. Johnson General Hospital	Texas
Ben Taub General Hospital	Texas

Clear Lake Regional Medical Center	Texas
University of Texas Southwestern Med. Ctr. Dallas	Texas
Kingwood Medical Center	Texas
Lake Pointe Medical Center	Texas
Cypress Fairbanks Medical Center Hospital	Texas
CHRISTUS Southeast Texas Hospital	Texas
Timpanogos Regional Hospital	Utah
Children's Hospital of the King's Daughters	Virginia
Inova Fair Oaks Hospital	Virginia
Centra Health, Virginia Baptist Hospital	Virginia
University of Virginia	Virginia
Children's Hosp of Richmond at VCU	Virginia
Johnston-Willis Hospital	Virginia
CJW Medical Center, Chippenham Campus	Virginia
Spotsylvania Regional Medical Center	Virginia
Mary Washington Hospital	Virginia
Virginia Hospital Center	Virginia
Inova Loudoun Hospital	Virginia
Deaconess Hospital	Washington
Providence Sacred Heart Medical Center & Children’s Hospital	Washington
Kadlec Regional Medical Center	Washington
UW Medicine - Valley Medical Center	Washington
Legacy Salmon Creek Hospital	Washington
PeaceHealth Southwest Medical Center	Washington
Evergreen Health	Washington
Overlake Hospital Medical Center	Washington
Providence Regional Medical Center Everett	Washington
MultiCare Health System - Tacoma General Hospital	Washington
West Virginia University School of Medicine	West Virginia
St. Joseph Hospital-Marshfield Clinic	Wisconsin

SSM Health St. Mary's Hospital Madison	Wisconsin
Ascension - St. Joseph's Hospital	Wisconsin
Aurora Sinai Medical Center	Wisconsin
Columbia St. Mary's Hospital	Wisconsin
Ascension All Saints Hospital	Wisconsin
Gundersen Lutheran Medical Center	Wisconsin
St. Vincent Hospital	Wisconsin
Children's Hospital of Wisconsin, Fox Valley	Wisconsin
Affinity NICU at St. Elizabeth Hospital	Wisconsin
Meriter Hospital	Wisconsin
Aurora Baycare Medical Center	Wisconsin
Aspirus Wausau Hospital	Wisconsin
Aurora Women's Pavilion	Wisconsin
